# Quantifying the dyadic interaction: A methodological framework for conducting observational moment-by-moment research

**DOI:** 10.3758/s13428-026-03162-2

**Published:** 2026-08-27

**Authors:** Diego Fernández-Regueras, M. Cristina Guerrero-Escagedo, Ana Calero-Elvira, Eduardo Estrada

**Affiliations:** 1 Departamento Psicología Biológica y de La Salud, Universidad Autónoma de Madrid, Madrid, Spain; 2 Departamento Psicología Social y Metodología, Universidad Autónoma de Madrid, Calle Ivan Pavlov, 6. Despacho A22, 28049 Madrid, Spain

**Keywords:** Dyadic data analysis, Interdependent behavior, Systematic observation, Observational data, *R* computing language, Therapeutic alliance

## Abstract

Systematic observation is a valuable method for studying interactive phenomena in dyads, as it captures sequences of behavior, potentially enhancing validity. However, observational studies remain underutilized compared with other tools, such as self-report questionnaires, because they require time and resources. This work aims to facilitate observational research by providing a step-by-step guide for preprocessing and exploring data from systematic observations, enabling flexible data handling beyond traditional sequential analyses. To illustrate the guide’s application, we used a dataset of 154 psychotherapy sessions collected longitudinally across different treatment phases from 32 patient–therapist dyads. We coded sessions using the Therapeutic Relationship Coding System (TRCS), which captures moment-by-moment verbal interactions relevant to the therapeutic alliance. Post-session questionnaire data were collected using the Working Alliance Inventory (WAI). An *R* script and a set of functions were developed to assist researchers with data preprocessing and to generate descriptive visualizations addressing the following questions: (1) *What behaviors (and sequences of interacting behaviors) are more frequent?*; (2) *How do behaviors evolve over time*; (3) *Do behavioral changes differ across groups?* After applying our proposed analytical tools, we interpret our results in light of the dominant theoretical framework on the therapeutic alliance.

## Introduction

Many psychological phenomena cannot be understood without paying attention to the temporal interdependencies between the variables involved (Bateson & Martin, 2021). When the analysis focuses on a dyad, the *interaction* between the two members can be defined as their dynamic exchanges, in which their actions influence and shape each other (Wagner, 1994). Psychotherapy is an excellent example, as it inherently involves interaction between therapists and clients. Understanding the therapeutic process requires shifting the unit of analysis from the individual to joint patterns of behavior (Hategeka et al., 2021).

Other domains that can benefit from this perspective shift include education and learning environments (Zheng et al., 2024), work/organizational behavior (Lehmann-Willenbrock, 2025), ethology (Amici et al., 2024), or caregiving behavior (Kosie & Lew-Williams, 2024), among many others. All of these involve two or more individuals whose actions influence one another, ultimately leading to an outcome different from the mere sum of its parts (Wagner, 1994).

Observational methodology involves direct coding of behavior within relevant contexts. By capturing behaviors as they occur, observational data can provide high-quality information and potentially achieve higher external validity than retrospective self-report measures (Baumeister et al., 2007).

Despite its advantages, observational methodology has been underutilized, in part because it is costly in time and resources (Atzil-Slonim et al., 2024; Robbins et al., 2024). Self-report measures are usually favored because they allow researchers to recruit larger samples in shorter time periods (Sassenberg & Ditrich, 2019). However, recent developments in large language models (LLMs) and other artificial intelligence models are paving the way for automatic coding of speech content features in psychological research (Aafjes-van Doorn et al., 2021; Eberhardt et al., 2025).

Among observational methods, systematic observation is particularly useful for studying dyadic interactions, as it allows capturing real-time dynamics in a structured manner (Bateson & Martin, 2021), making it well suited to the study of dyadic interactions. When using systematic observation, the basic unit of analysis is the *observational session*, which consists of a *sequence* of coded events—typically behaviors—emitted by the individuals under study, often termed *agents*. The so-called molecular analysis of interaction involves segmenting this sequence into predefined units of at least two events or behaviors. Sequential analyses, in turn, typically aim to identify contingency patterns between events (Bakeman & Quera, 2011). It therefore addresses the question of whether a specific sequence of events—often two, sometimes three or four—occurs at a frequency greater than chance.

A large part of the literature on sequential analysis has used conventional framework termed the *Sequential Data Interchange Standard* (*SDIS*), and a statistical software called *Generalized Sequential Querier* (*GSEQ*), for conducting contingency analyses (Bakeman & Quera, 2011). The most frequent statistical tools include joint frequencies of behaviors occurring in sequence, chi-square tests, and log-linear analysis (Bakeman & Quera, 2009). Recently, the *R* package *ConNEcT* has been developed to perform similar analyses to GSEQ, offering some additional features (Bodner & Ceulemans, 2023).

One important challenge in observational studies is the limited size of the samples available. This is often due to the time-consuming and resource-intensive nature of these studies (Atzil-Slonim et al., 2024; Robbins et al., 2024). Additionally, multi-event sequences are rarer than single events, which reduces the effective sample of observable sequences. However, certain events can significantly influence the trajectory and outcomes of the observed interaction, even if they occur only once or a few times across all the analyzed sessions. For instance, in the context of psychological therapy, if a therapist responds to a client’s explanation of their behavior with hostility, even if only once, this may undermine subsequent therapeutic collaboration (Muran & Eubanks, 2020). In other words, some phenomena naturally occur more frequently than others, but their frequency does not necessarily determine their impact; rather, their influence depends on occurring at the right moment and in the right context (Levy Chajmovic & Tishby, 2025). Furthermore, because dyadic interactions develop over the course of therapy, researchers can study them only through repeated measures across sessions (Crits-Christoph & Gibbons, 2021; Zilcha-Mano & Fisher, 2022). This highlights the importance of studying behavior sequences and how they change over the course of therapy.

## The present work

 Building on these challenges, this paper provides researchers with a step-by-step methodological guide for studying interactive processes in dyads (Note: for conciseness, we use the term *behavior* to refer to both individual behaviors and short sequences of behaviors throughout). We propose a set of procedures and tools for addressing the following questions:

1. *What behaviors are more frequent?* Overall frequency of occurrence can provide meaningful insights in specific research contexts. This guide introduces several tools for examining frequency patterns in observational data.
2. *How do the frequencies of behaviors change across sessions?* We present several tools to track the trajectories of *behaviors* over time while controlling for overall frequency differences, to enable a clearer understanding of behavioral dynamics (considering that some phenomena are inherently more frequent than others, regardless of their importance).
3. *Do changes in behaviors differ across groups?* Different predefined groups of dyads (e.g., applying intervention A vs. intervention B) may show differences in the occurrence and evolution of behaviors and frequencies, which in turn may be associated to different therapy outcomes. We provide tools to describe, quantify, and visualize the trajectories observed in each group. These tools include both plots for visual inspection and computations of the sample size, mean, median, standard deviation, and 5th and 95th percentiles of each behavior or sequence for every group and treatment phase. We will illustrate how to apply the proposed procedures with an empirical dataset comprising interactions between a sample of therapist-client dyads. Importantly, our proposed framework can be applied to other research domains in which interactive dyadic processes are relevant, such as education and psychological development (e.g., parent–child interactions), organizational behavior, ethology, or caregiving behavior.

All the steps described for preprocessing and analyzing the data, including the behavioral coding’s files and R code, are available on an accompanying website to ensure that other researchers can use the functions: https://dfernandezregueras.github.io/step-by-step-guide/

For citation purposes, we ask that users of the functions cite the present manuscript.

## Description of the illustrative empirical data

As an illustrative example, we will use an observational dataset including 32 clinical cases, coded longitudinally across different phases of psychotherapy. The dataset includes complete coding of 154 therapy sessions, analyzed using the *Therapeutic Relationship Coding System* (TRCS; Guerrero-Escagedo et al., 2025). This is a coding system designed to code moment-by-moment verbal behaviors exhibited by therapists and clients and can be used to categorize a broad range of therapeutic alliance-related behaviors (Guerrero-Escagedo et al., 2026). The *therapeutic alliance* is a relational construct referring to a style of collaboration between therapists and clients characterized by care and mutual respect (Horvath et al., 2011).

Two expert observers coded the sessions. At the time of coding, both were licensed psychotherapists pursuing their PhDs and had more than 5 years of experience in observational coding using the TRCS and other coding schemes. Event duration varied depending on the TRCS category, ranging from brief interjections to speeches lasting several minutes. Consecutive instances of the same behavior could be coded as separate events. Detailed information about the TRCS can be found in Guerrero-Escagedo et al. (2025). The observation yielded a total of 108,192 coded behaviors (in line with the literature, we will refer to them as *codings*). Agreement between observers, computed randomly in 10% of the sessions, was good to excellent (Cohen’s kappa =.66–.83; *observer accuracy* = 82–92%) (Bakeman, 2023; Bakeman & Quera, 2011). Because observational coding is time- and resource-intensive, we considered this 10% subsample sufficient under our coding protocol, which required further one-by-one review if reliability fell below acceptable levels.

For each of the 32 dyads, up to five sessions were coded, randomly chosen but representing different phases of psychological therapy: one session from the initial assessment phase, one from the treatment planning phase, and between one and three from the intervention phase. The number of coded intervention sessions depended on the total number of intervention sessions for each case: one coded session for cases with 1–3 intervention sessions (short therapy duration), two coded sessions for cases with 2–7 sessions (medium therapy duration), or three coded sessions for cases with > 7 sessions (long therapy duration). Importantly, we did not summarize the intervention phase by pooling multiple sessions within a dyad; instead, we analyzed each coded intervention session separately and classified it by phase (beginning, middle, or end). Thus, in shorter cases such as dropouts, we included only the phase(s) actually observed in that case.

Post-session questionnaire data were also collected using the *Working Alliance Inventory* (WAI; Horvath & Greenberg, 1989), which assesses the self-reported quality of the therapeutic alliance. Clients responded to the complete questionnaire at three time points: (1) after the third session, (2) two sessions after the treatment planning session, and (3) at the end of therapy. Internal consistency was adequate in all subscales and time points (*α* =.89–.95; *ωₕ* =.89–.95). We created two groups, based on the mean therapeutic alliance score, as measured by the WAI across multiple time points during therapy. Specifically, the ten dyads with the highest and the ten with the lowest average scores were included in the “Good” and “Poor” alliance groups, respectively.

The illustrative sample comprised face-to-face psychotherapy sessions from the university clinic at the Autonomous University of Madrid (Spain), conducted using a cognitive–behavioral therapy (CBT) model. Case presentations were diverse, with a predominance of anxiety-related problems. Therapists were primarily early-career clinicians with one to three years of clinical experience. All participants identified as White. Demographic characteristics of the sample are reported in Table 1.

**Table 1:** Demographic data for the patients and therapist included in the illustrative empirical data

Variable	Category	
Clients’ demographics		
Age	*M* = 26.75 (*SD* = 11.90)	
Gender	Female	17 (53.12%)
	Male	15 (46.88%)
Diagnoses	Anxiety	20 (62.50%)
	Depression	6 (18.75%)
	Eating	2 (6.25%)
	Obsessive–compulsive	2 (6.25%)
	Attention deficit/hyperactivity	1 (3.12%)
	Trauma	1 (3.12%)
Therapists’ demographics		
Gender	Female	24 (75.00%)
	Male	8 (25.00%)
Experience (years)	*M* = 2.34 (*SD* = 2.39)	

**Fig. 1:**
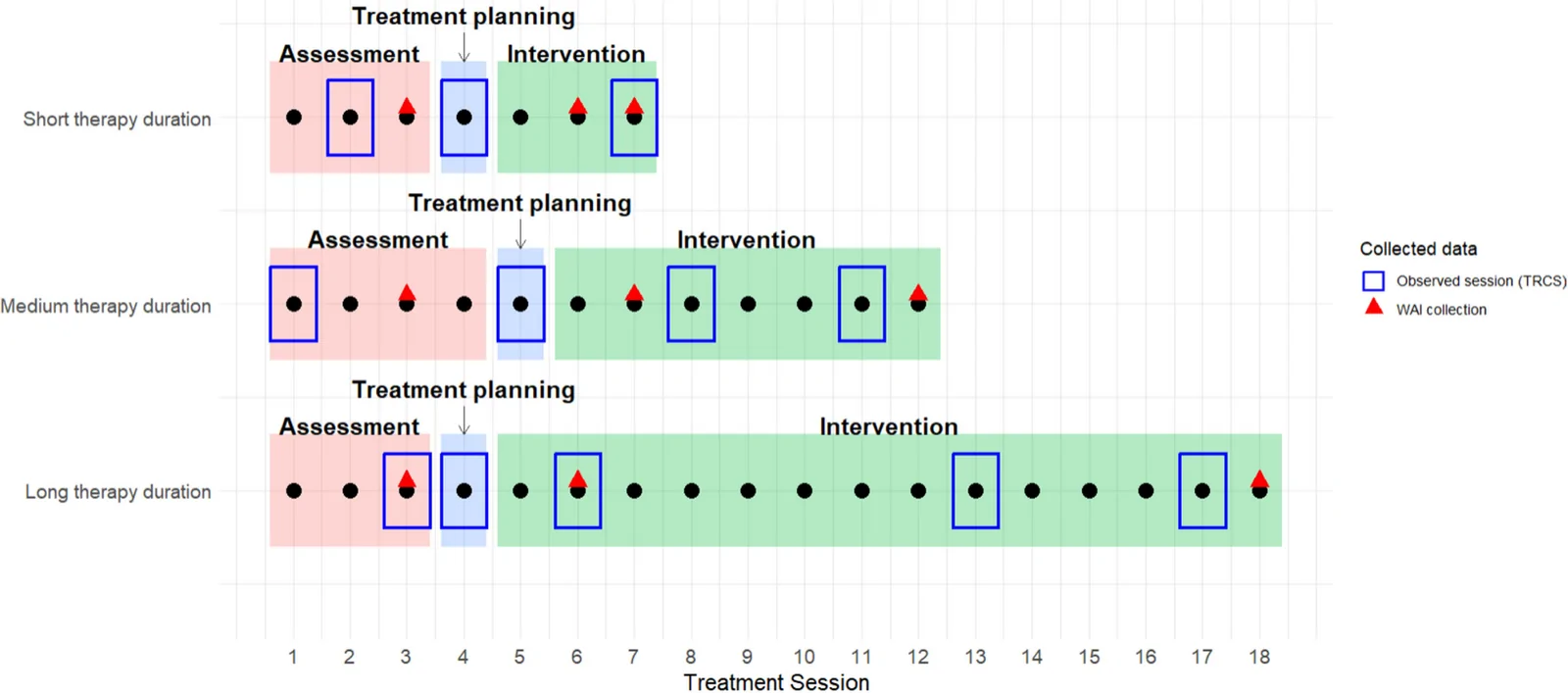
Data collection: temporal distribution of treatment phases, observed sessions, and questionnaire administration in three exemplary dyads of short, medium, and long therapy duration. Note: *TRCS* = Therapeutic Relationship Coding System, *WAI* = Working Alliance Inventory

Figure 1 depicts the temporal distribution of the coded sessions and the questionnaire evaluations in three example dyads of short, medium, and long therapy duration.

Following standard recommendations on observational studies (Bakeman & Quera, 2011), we now describe the nature of our data to clarify the scope of applicability for the guidelines presented in this paper.

- The TRCS is a *coding scheme* consisting of a set of labels (i.e., categories or codes) assigned to specific events (*behaviors*). It codes whether the event occurred. It also allows specification of the order in which the behaviors of interest appeared.
- The recording strategy employed was *event-contingent recording* (also called *untimed-event recording*, e.g. Bakeman & Quera, 2011), in which the duration of the events is not coded. This implies that, for example, in a one-minute segment, it is possible to register as many *behaviors* as needed.
- The TRCS was applied to *recorded sessions*.
- The sessions were coded using *computer-assisted coding*. We employed *The Observer XT 16* (Noldus Information Technology, 2022). Computer-assisted coding is particularly beneficial for researchers working with extensive coding schemes. Cohen’s kappa for rater reliability was computed using this software, while observer accuracy was assessed with *KappaAcc* (Bakeman, 2023).

We note that the step-by-step guide presented in this paper can be readily applied to any data consisting of a continuous sequence of behaviors, provided that the order of behaviors is preserved row by row and that the data include both members of the dyad.

The data collection was reviewed and approved by the Research Ethics Committee of the *Universidad Autónoma de Madrid* (approval code CEI-133–2788). Written informed consent was obtained from both therapists and clients, in compliance with the applicable legislation.

Table 2 provides a schematic overview of the steps that we will explain in the following sections, along with the tasks performed within each step.

**Table 2:** Overview of the steps in the procedure; available in the accompanying website https://dfernandezregueras.github.io/step-by-step-guide/

Step	Tasks involved	Section in the accompanying website and available functions
A. Data extraction from coding software	• Export observational data in a *R* readable format (e.g.,.xlsx or.csv)	
B. Preprocessing (observational data)	• Load the observational data in *R* • Merge different files in which the data may be stored	1.1
	• Assign to each session the corresponding levels in the following variables: a. *Phase of therapy*: to allow longitudinal analyses b. *ID*: to identify each dyad coded in various moments c. *Group*: to allow comparisons between groups of dyads	1.2
	• Permanently group behaviors that are theoretically similar into a single coding, for subsequent analyses	1.3 • permanent_group()
C. Descriptive statistics	• Obtain descriptive statistics for the behaviors of interest • Generate plots to examine the distribution of the behaviors of interest • Compare the descriptive statistics/plots across different groups	2.1 • sequence_descriptives()
D. *What behaviors are more frequent?*	• Generate heatmaps for different behaviors to compare their relative or absolute frequencies across treatment phases • Customize output to address specific research questions (e.g., order the behaviors based on their frequency) • Compare the heatmaps generated for different groups	3.1 • sequence_heatmap()
E. *How do the frequencies of behaviors change across sessions?*	• Obtain the distribution of the behaviors across treatment phases using a standardized metric (i.e., regardless of overall frequencies of each one) • Check the best-fitting shape of the longitudinal change (linear, quadratic or cubic, based on *AIC*) • Compare the shape of change for different behaviors	3.2 • sequence_trajectory()
F. *Do changes in behaviors differ across groups?*	• Compare the different shapes of change, in a standardized metric, across different groups of IDs • Check the best-fitting shape of the longitudinal change (linear, quadratic or cubic, based on *AIC*) for each group	3.3 • sequence_trajectory()

## Data preprocessing

**Fig. 2:**
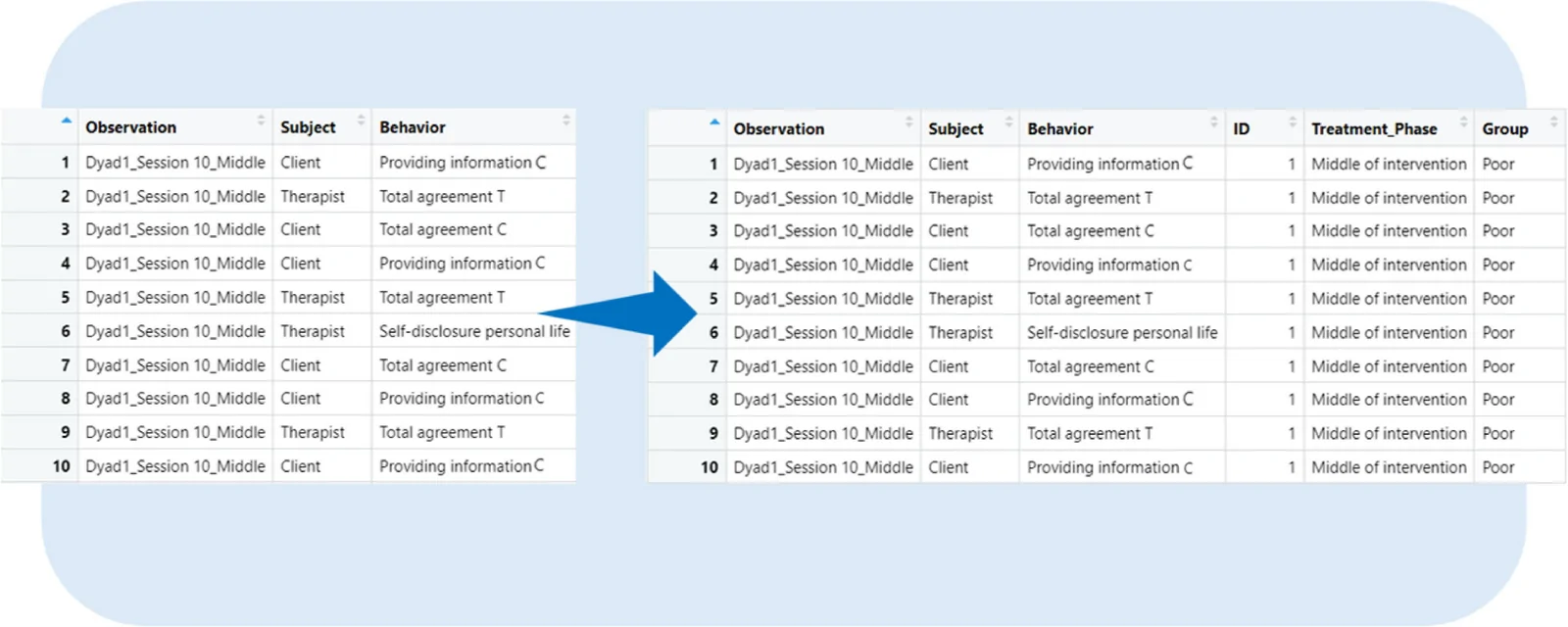
Process of addition of new variables: (1) Variables provided by The Observer XT; (2) variables ID, treatment phase and group added

 Irrespective of the chosen coding strategy, data must be exported to a spreadsheet format that can be loaded in *R* for statistical analyses. Most common coding programs can export coding data into various formats (e.g.,.xlsx,.txt). In this work, we used data coded through *The Observer XT v. 16* (Noldus ©); however, the functions are compatible with data exported from other coding platforms, including free and open-source alternatives such as BORIS (Friard & Gamba, 2016), CowLog (Hänninen & Pastell, 2009), or Datavyu (Datavyu Team, 2014), provided that the data are arranged in a spreadsheet-compatible format. The dataset used in this step must include the following key variables, in order to apply our preprocessing functions: (1) *Behavior*: contains the list of coded events; (2) *Session* (alternatively called *Observation*, e.g., in The Observer XT): each different value includes all the behaviors recorded within a single session (e.g., a therapy session); (3) *Agent* (alternatively called *Subject* in The Observer XT): identifies who emitted each behavior. Note that this dataset is structured in long format, i.e., each row contains a single *Behavior*, and the same *Observation* and *Agent* can appear repeatedly, in as many rows as needed. The left section of Fig. 2 depicts this data structure.

The first step involves loading the observational data extracted from the coding procedure. Because observation is a time-consuming process, it is common for multiple researchers to be involved or for a single researcher to conduct observations at different coding sessions. As a result, data may be stored in multiple files. It is recommended to use the three variables described above (*Behavior*, *Observation*, and *Agent*), which are often provided by the coding software, to merge data into a single file in which the repeated measures are structured in long format. Section 1.1 in our supplementary website (https://dfernandezregueras.github.io/step-by-step-guide/) provides functions to perform this task. Once the initial dataset is prepared, several variables must be added for subsequent analyses: (1) *Therapy phase*: given the longitudinal nature of data, which involves within-person repeated observational sessions across treatment, it is crucial to categorize each session according to the therapy phase it belongs to; (2) *ID*: a unique identifier for each patient-therapist dyad, held constant for the different sessions and therapy phases; (3) *Group*: if researchers wish to classify IDs into higher-level categories (e.g., based on therapeutic alliance quality groups, age ranges, or types of psychological problem). The right section of Fig. 2 shows a subset of the dataset to illustrate its structure and the process of addition of the new variables. Section 1.2 of the supplementary website (https://dfernandezregueras.github.io/step-by-step-guide/) provides an example of how to create these variables.

A final preprocessing step involves the optional *permanent grouping of behaviors*. For example, in the Spanish sample used in our motivating example, cultural norms may lead “*Partial Agreement*” and “*Disagreement*” to reflect similar relational dynamics. In such cases, you can merge these categories from the outset. In contrast, in other cultures where direct disagreement is more socially acceptable, retaining finer distinctions may be preferable, depending on the research objectives. To support this flexibility, we developed the permanent_group() function, which lets users permanently consolidate behavior categories when appropriate. An example of its use is provided in Sect. 1.3 of the supplementary website https://dfernandezregueras.github.io/step-by-step-guide/

## Descriptive statistics and visualization

**Fig. 3:**
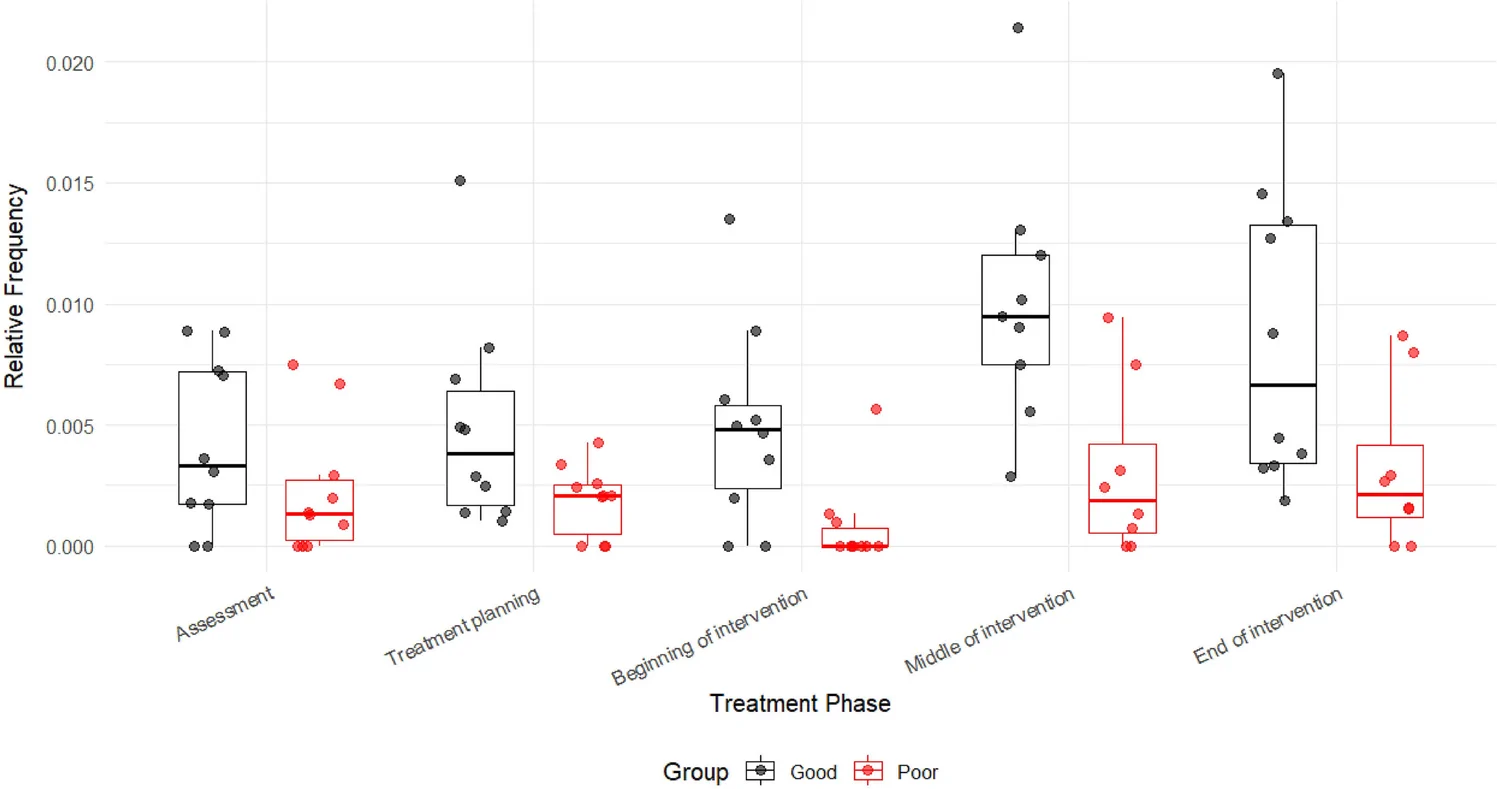
Boxplot generated by the function sequence_descriptives() for the sequence “Empathy + Present success (therapist)—> Total agreement (client)” across treatment phases and comparing alliance quality groups (good vs. poor)

 Once the final dataset has been obtained, the next step is to examine the behaviors of interest. The function sequence_descriptives() computes descriptive statistics of the selected behaviors for each treatment phase and *for all the dyads* in the sample, either together or separated by groups. These statistics include the number of observation sessions in which the selected behaviors appear at least once, mean, median, standard deviation, 5th and 95th percentiles, skewness and its standard error, and kurtosis. These can be computed using absolute or relative frequencies per observation session, with the latter calculated by dividing the number of target behaviors in a session by the total number of behaviors coded in that session. This standardization allows examining how prevalent the behavior of interest is, taking into account that the total number of *codings* may vary greatly between different sessions and different dyads. In addition, the Shapiro–Wilk test for normality is conducted as it has been shown to be particularly robust for small sample sizes (LaFromboise, 2024). A boxplot is also generated to facilitate visual assessment of the data distribution. Table 3 and Fig. 3 depict an example of the descriptive table and boxplot for a the behavioral sequence *“Empathy* + *Present success (therapist)—*> *Total agreement (client)”* in all phases of therapy separating alliance quality groups (“Good” and “Poor”). See Sect. 2.1 of the supplementary website for more examples computed with various behaviors https://dfernandezregueras.github.io/step-by-step-guide/

**Table 3:** Descriptive statistics for the relative frequencies for the sequence “Empathy + Present success (therapist)—> Total agreement (client)” across all treatment phases and comparing alliance quality groups (good vs. poor)

Group	Treatment phase	*N*	*n*	*M*	*Mdn*	*SD*	5th Perc	95th Perc	*Skw*	*SE*	*Kur*	Shapiro *p*	Normality
Good	Assessment	10	10	.004	.003	.003	<.001	.009	.160	.774	– 1.796	.149	Normal
	Treatment planning	10	10	.005	.004	.001	.001	.012	1.151	.774	.274	.040	Not normal
	Beginning of intervention	10	10	.005	.005	.004	<.001	.011	.658	.774	–.477	.382	Normal
	Middle of intervention	9	9	.010	.009	.005	.004	.018	.746	.816	–.158	.471	Normal
	End of intervention	10	10	.008	.006	.006	.002	.017	.411	.774	– 1.506	.159	Normal
Poor	Assessment	10	10	.002	.001	.002	<.001	.007	.950	.774	–.773	.012	Not normal
	Treatment planning	10	10	.002	.002	.001	<.001	.004	–.088	.774	– 1.372	– 179	Normal
	Beginning of intervention	10	10	.001	<.001	.001	<.001	.004	1.988	.774	2.635	<.001	Not normal
	Middle of intervention	8	8	.003	.002	.003	<.001	.009	.756	.866	– 1.222	.065	Normal
	End of intervention	8	8	.003	.002	.003	<.001	.008	.680	.866	– 1.362	.051	Normal

Table generated by the function sequence_descriptives()*. N* = total number of sessions included in the treatment phase; *n* = number of sessions in which the target behavior appears at least once; *M* = mean relative frequency (i.e., on average of the total number of codes for each session, what proportion corresponds to the behavior of interest); *Mdn* = median; *SD* = standard deviation; 5th Perc. = *5*th percentile; 95th Perc. = *95*th percentile; *Skw.* = skewness; *SE* = *skewness*’ standard error; *Kur* = kurtosis; Shapiro* p* = Shapiro–Wilk test’s *p* value

Table 3 presents the descriptive statistics for the behavior sequence “*Empathy* + *Present success (therapist)—*> *Total agreement (client)*” for all treatment phases using relative frequencies and comparing alliance groups “good vs. poor”, based on their mean WAI score. Focusing on the “Assessment” phase and the “Good” alliance group as an example, out of the *N* = 10 sessions within this phase, all (*n* = 10) contained at least one coding of the target sequence. The mean relative frequency across all sessions was *M* =.004, indicating that, on average, the sequence accounted for approximately 0.4% of the total sequences per session in this phase. The median was *Mdn* = *.*003, lower than the mean and consistent with a positive skewness (*Skw* =.160). The fifth and ninety-fifth percentiles were less than.001 and.009, respectively. This implies that, in 90% of the sessions, the sequence of interest represented between less than.01% and 0.90% of all behaviors recorded. The Shapiro–Wilk test was not significant (*p* = *.*149), which implies that normality can be assumed. Figure 3 displays the distribution of relative frequencies for this sequence across all sessions. Consistent with the descriptive statistics depicted in Table 3, data appear to approximate a normal distribution in the “Assessment” phase and “Good” alliance group.

The descriptive statistics provided by the function sequence_descriptives() allow a quick and exhaustive exploration of the distribution of one or more *behaviors* of interest. This descriptive analysis is very informative, as it allows answering questions about various aspects of the therapeutic interaction (e.g., How prevalent is the behavior? How heterogeneous the sample is?). Furthermore, the function includes a normality check, as normality is one of the assumptions of linear models and parametric tests that researchers may want to perform subsequently, and it also informs the choice of interval estimator in sequence_trajectory() (see below). One example of such linear modeling is provided in the section “How do *behaviors* evolve over time?”.

According to Table 3 and Fig. 3, the sequence in which the therapist emits “*Empathy* + *Present success”* and the client responds with “*Total agreement”* was more frequent in the group of dyads with good therapeutic alliance, across all therapy phases, with the difference between groups increasing in the middle and final phases of the intervention. Because the alliance groups were defined from WAI scores, a client self-report of perceived alliance quality, whereas the frequencies come from TRCS observational coding, this pattern reflects convergence across two measurement perspectives rather than a comparison between independent constructs. We return to the implications of this overlap in the Discussion.

Because each session in our dataset contains a different number of coded behaviors—due to factors such as variability in therapist or client talkativeness, descriptive statistics are computed using the relative frequencies of behaviors and sequences per therapy session. I.e., the obtained frequencies are expressed as a proportion of the total number of behaviors in the session. However, the function can also provide absolute frequencies. This may be useful if the coding scheme implies recording a fixed number of behaviors per observation session (e.g., interval recording schemes where the most prevalent behavior exhibited by each agent is coded in each interval).

The function sequence_descriptives(), as well as sequence_heatmap(), and sequence_trajectory() (described later) allow grouping the behaviors of interest flexibly for analysis without modifying the original dataset by using the ‘ + ’ operator. The results can also be displayed either for the entire dataset or separated by groups based on the *Group* variable.

## What *behaviors* are more frequent?

**Fig. 4:**
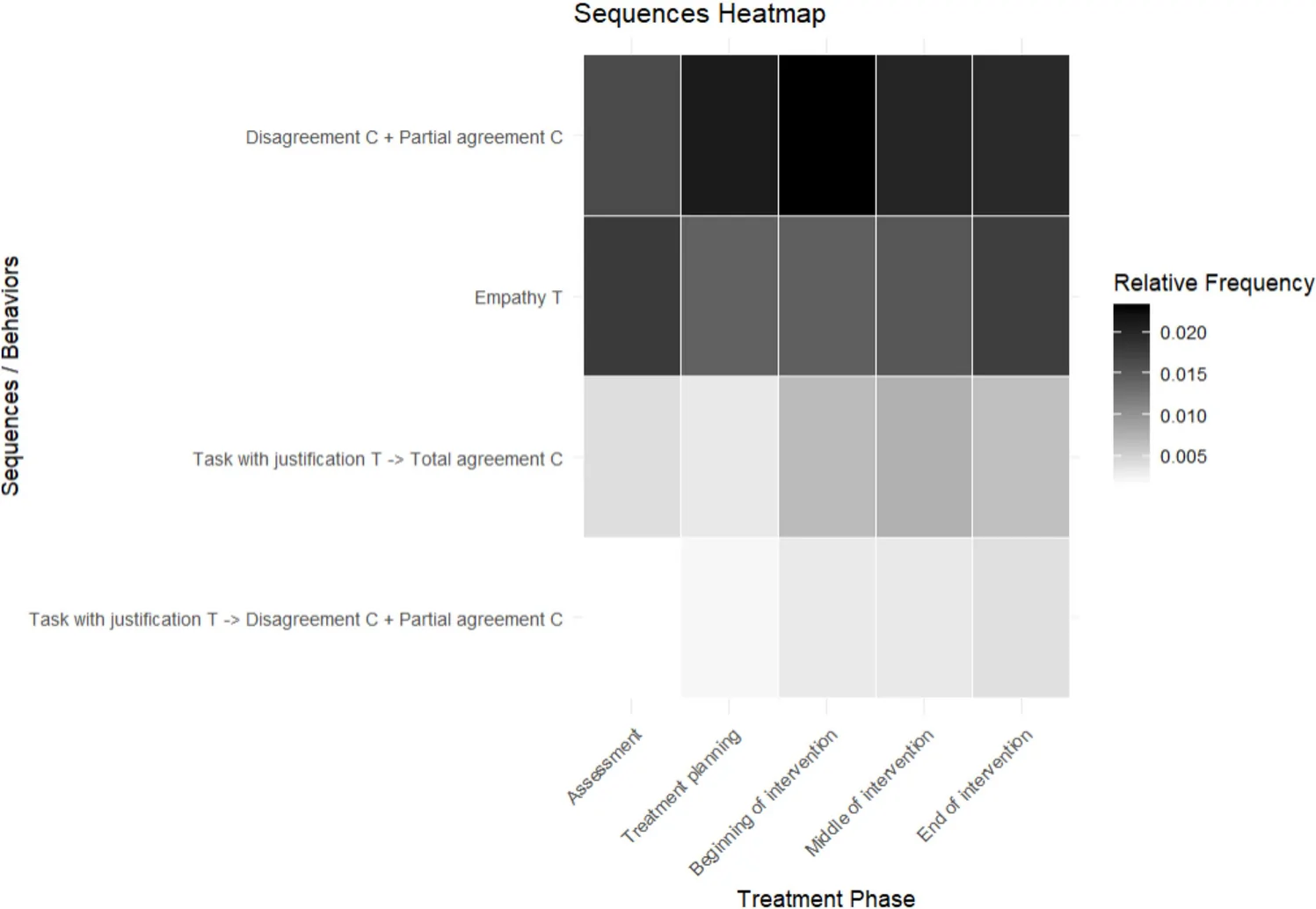
Heatmap generated with the function sequence_heatmap() comparing the relative frequency of various behaviors across treatment phases and ordered by their total frequency (for the whole sample). *Note: T* = behavior emitted by the therapist; *C* = behavior emitted by the client

To answer this research question, we developed the function sequence_heatmap(). It allows generating heatmap plots for the selected behaviors. This function offers flexible customization options, including choosing between absolute or relative frequencies, ordering behaviors in the plot, and comparing different groups of dyads. Figure 4 depicts a plot generated with this function.

To facilitate the interpretation, we apply a frequency normalization similar to the one applied in the previous section. The sequence_heatmap() function calculates the frequency of the target *behaviors* within each treatment phase, across all sessions and dyads. When using relative frequency (i.e., freq_type = “relative”), for a given *behavior*, the function divides the number of times it appears in each phase by the total number of behaviors (of any type) coded in that phase for that dyad. As a result, the relative frequencies reflect how prominent a specific behavior is within each phase, relative to the overall *codings* in that phase and dyad. For example, a relative frequency of 0.25 in the assessment phase indicates that one quarter of the total behaviors in that phase correspond to the target behavior. Nevertheless, researchers can opt to compute absolute frequencies per treatment phase (setting the argument freq_type = “absolute”). 

In Fig. 4, each square represents one behavior in one phase of therapy. Darker shading indicates higher relative frequency. Among the behaviors shown in the figure, the most frequent one across all treatment phases is the group “Partial agreement + Disagreement”. These are behaviors emitted by the client. The darker shading implies that these behaviors appear more often in the “Treatment planning” and “Beginning of intervention” phases. This is likely because these sessions involve negotiation of treatment goals between therapist and client. In contrast, such behaviors are less frequent in the “Assessment” phase—where clients primarily describe their problems with limited therapist input—and in the middle and final phases of intervention, when clients may have become more accustomed to the intervention and experience fewer reasons to disagree.

Empathy (emitted by therapists) is the second most frequent behavior in Fig. 4. Its lower frequency compared to disagreement-related behaviors is expected, as disagreement may arise in response to various therapist verbalizations (e.g., suggesting tasks or goals, recalling prior information or making clarifications). However, therapist expressions of empathy tend to occur in more specific contexts, such as when clients express emotional distress. Interestingly, the distribution of “Empathy” across phases peaks during the “Assessment” and “End of therapy”, aligning with the therapeutic focus on these sessions. During “Assessment”, therapists aim to build alliance and understand clients’ concerns without yet introducing therapeutic tasks or goals. In the “End of therapy” phase, treatment goals have generally been addressed, creating space for empathic reflection on change. During the intermediate task and goal-focused phases, empathic responses might be relatively less prioritized in favor of goal and task-oriented behaviors.

The third and fourth most frequent *behaviors* in Fig. 4 are both sequences. As expected, they are less common than individual behaviors, since they depend on the co-occurrence of multiple events. “Task without explanation (therapist) → Total agreement (client)” is more frequent than “Task without explanation (therapist) → Partial agreement + Disagreement (client)”, which is consistent with expectations for effective therapeutic processes. Both sequences show higher frequencies during the three intervention phases, where therapeutic tasks are more commonly assigned. In contrast, the “Assessment” and “Treatment planning” phases primarily focus on understanding the client’s issues and establishing treatment goals, resulting in fewer task-related exchanges.

The longitudinal changes observed through this function can be very informative for designing future studies. For example, researchers interested in client disagreement might focus on “Treatment planning” and the initial intervention sessions, where these behaviors are more frequent because of the emphasis on negotiating goals. Conversely, studies examining sequences involving tasks and client responses may benefit from concentrating on the intervention phases, where such sequences are more frequent. In contrast, investigating these patterns in earlier phases may yield limited findings because task-related exchanges are relatively rare at that stage. Section 3.1 of the supplementary website (https://dfernandezregueras.github.io/step-by-step-guide/) provides both the function and an example of its use.

## How do the frequencies of behaviors change across sessions?

To examine patterns of change across treatment phases, the next step is to visualize trajectories of the *behaviors*. As previously, we used relative frequencies because the clinical relevance of certain phenomena depends more on their temporal occurrence than their overall frequency. However, note that the normalization applied here to compute relative frequencies is different from that of the two previous sections, since it is intended to answer a different question.

The function sequence_trajectory() calculates the absolute frequency of specified *behaviors* for each dyad (ID) within each phase. Then computes the relative frequencies by dividing the phase-specific counts by the dyad’s total occurrences of that *behavior* across all phases. I.e., the total number of *behaviors* in the category of interest emitted by the dyad under study is rescaled to 1, and therefore the number of *behaviors* in each phase is rescaled accordingly and expressed as a proportion. For example, a relative frequency of 0.25 in the assessment phase indicates that one quarter of the total *behaviors* of that type were emitted during that phase. This normalization enables comparison of trajectories between variables with differing absolute frequencies (as some naturally occur more frequently than others). As in previous steps, the *behaviors* can be flexibly grouped using the ‘ + ’ operator. The output displays both the individual trajectories and the average group trajectory.

Additionally, the function fits three models to examine the functional shape of the trajectory: linear, quadratic, and cubic. The function fits regularized Bayesian linear mixed-effects models (BLMMs) using the package *blme* (Chung et al., 2013). Random intercepts (1 | ID) are included to account for between-individual stable differences. The best-fitting function, among the ones specified by the researcher, is selected based on the Akaike information criteria (*AIC*), though Bayesian (*BIC*) is also reported. The empirical and model-implied means are plotted for visual inspection in blue and red lines, respectively. Error bars show 95% confidence intervals for the phase mean. Because the dyad-level values contributing to each phase mean is typically small, these are computed by default from the *t* distribution, as *M* ± *t*(.975, *n*—1) × *SE*. When the distribution of relative frequencies within a phase departs markedly from normality, bootstrap percentile intervals can be requested instead through the ci_method argument. This function is intended as an exploratory tool to summarize broad temporal patterns in observational dyadic data. The proposed trajectories provide informative descriptions of change rather than exhaustive model fitting. Approaches such as hidden Markov models, stochastic differential equation models, or network-based methods require many repeated measurements per unit to identify their parameters, and are therefore not estimable in designs of the kind illustrated here, with few measurement occasions per dyad (five treatment phases) and modest samples. With five occasions, the cubic term already exhausts the available degrees of freedom, so the polynomial family implemented in shapes is effectively saturated for this design.

**Fig. 5:**
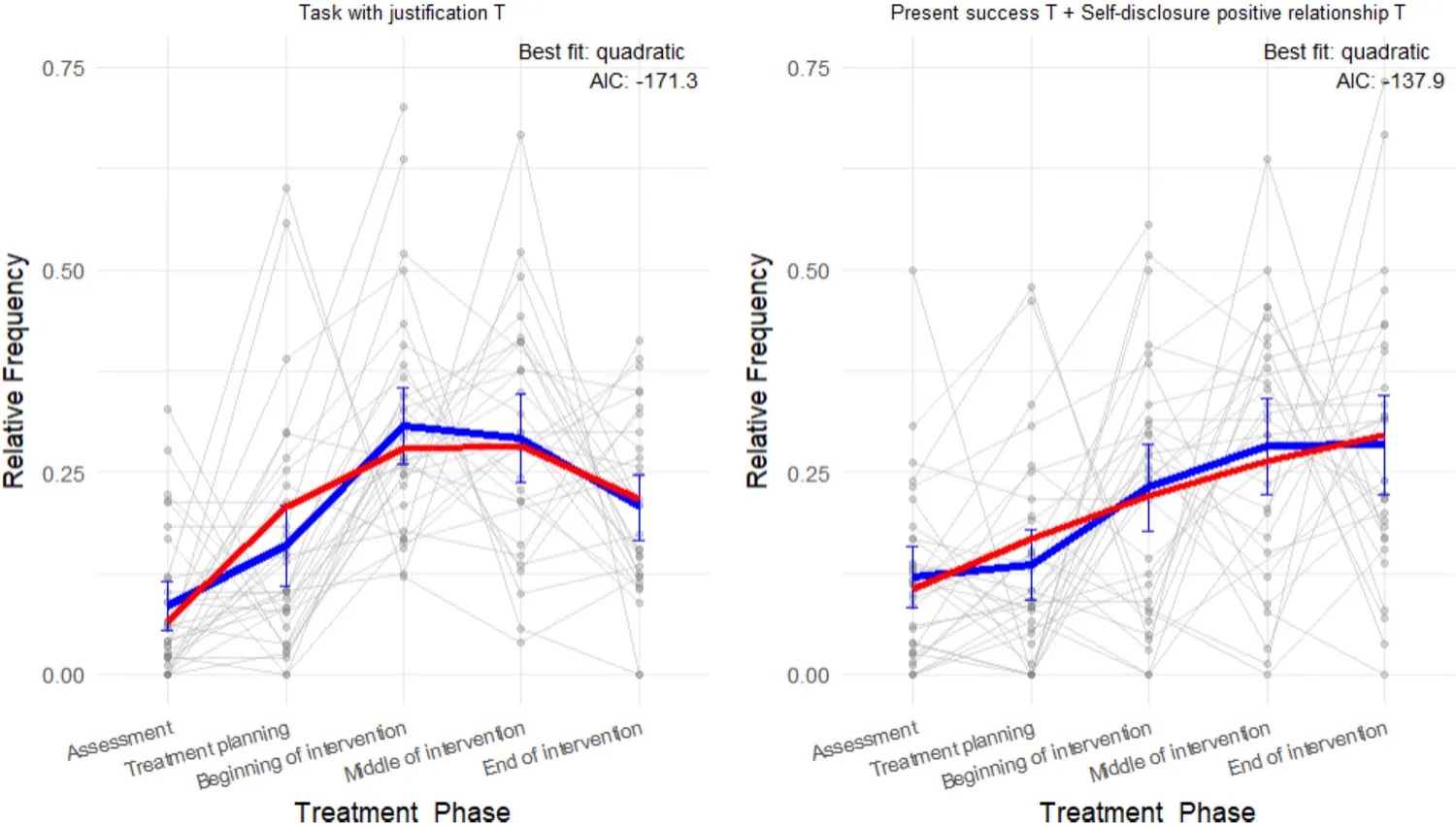
Plot generated with the function sequence_trajectory(), comparing the empirical and model-implied mean frequencies of the behaviors “Task/technique with an explanation” (*left panel*) and “Present success + Positive relationship self-disclosure” (*right panel*). *Note: T* = behavior emitted by the therapist; *C* = behavior emitted by the client. *Grey lines* = individual empirical frequencies; *blue line* = empirical mean; *red line* = model-implied mean. *Error bars* represent 95% confidence intervals, calculated as *M* ± *t*(.975, *n* – 1) × *SE*, where *n* is the number of dyads contributing data to that phase. The function sequence_trajectory() can alternatively compute bootstrap percentile intervals (ci_method = "bootstrap")

Figure 5 displays a plot generated with this function comparing the longitudinal distribution across treatment phases of two behavioral sequences: *“Task/technique with an explanation”* (left panel), and *“Present success* + *Positive relationship self-disclosure”* (right panel). Section 3.2 of the supplementary website (https://dfernandezregueras.github.io/step-by-step-guide/) provides the function description and another use example.

The left panel of Fig. 5 shows that the mean relative frequency of the therapist providing a “*task with explanation*” increases as therapy progresses, peaking during the “Beginning of intervention” and “Middle of intervention” phases, and then declining in the “End of intervention” phase. This pattern aligns with the therapeutic structure: during the “Assessment” phase, therapists primarily focus on building a therapeutic alliance and understanding the clients’ demands, so tasks are rarely introduced. Although task frequency increases slightly in the “Treatment planning”—marking the transition toward intervention—, the emphasis remains on negotiating treatment goals. In the “Beginning” and “Middle of intervention” phases, task assignment is more frequent, as tasks are a core tool in cognitive-behavioral therapy (CBT), facilitating skill generalization outside of the sessions. In the “End of intervention” phase, while still relevant, tasks are less frequent as most therapeutic goals have already been addressed. The best fit (according to *AIC*) is obtained by the cubic model, which implies two changes of tendency: a slight increase from the “Assessment” to the “Treatment planning” phase, a steeper rise during the early intervention phases, and a decline in the “End of intervention” phase.

The right panel of Fig. 5 illustrates the trajectory of the grouped behavior “*Present success* + *Self-disclosure of positive relationship*”, both emitted by the therapist. Frequencies increased across the early treatment phases, which is expected given that the alliance is developing and the client is progressively beginning active work toward treatment goals. This behavior stabilized at its maximum during the later stages of intervention—the “Middle” and “End of intervention” phases—likely reflecting the therapist’s acknowledgment of the client’s progress and the consolidation of the therapeutic alliance. The best fit (according to *AIC*) is obtained by the quadratic model, capturing an initial increase followed by stabilization in the final phases of treatment.

The standardized trajectories can help inform both research questions and clinical practices. The function sequence_heatmap() may also support this task by using relative frequencies. However, because it computes relative frequencies per session—comparing the target behavior to the rest of the behaviors in that session—some differences between phases might simply reflect higher frequencies of other behaviors, making the target behavior’s relative frequency appear lower. Standardizing each behavior based on its own total frequency across all sessions overcomes this limitation and offers a more accurate analysis of longitudinal changes. For example, the behavior “*Task with an explanation*” is more frequent during the beginning and middle of the intervention, suggesting that researchers might focus their research questions on those phases. In the case of “*Present success T* + *Self-disclosure of positive relationship T*”, researchers could explore its progressive increase during the early phases of therapy and how different rates of increase relate to other phenomena. Alternatively, they might focus on the middle and final phases of intervention, where the behavior appears more frequently, likely due to the therapeutic alliance being more developed and treatment goals having been largely accomplished.

## Do changes in *behaviors* differ across groups?

**Fig. 6:**
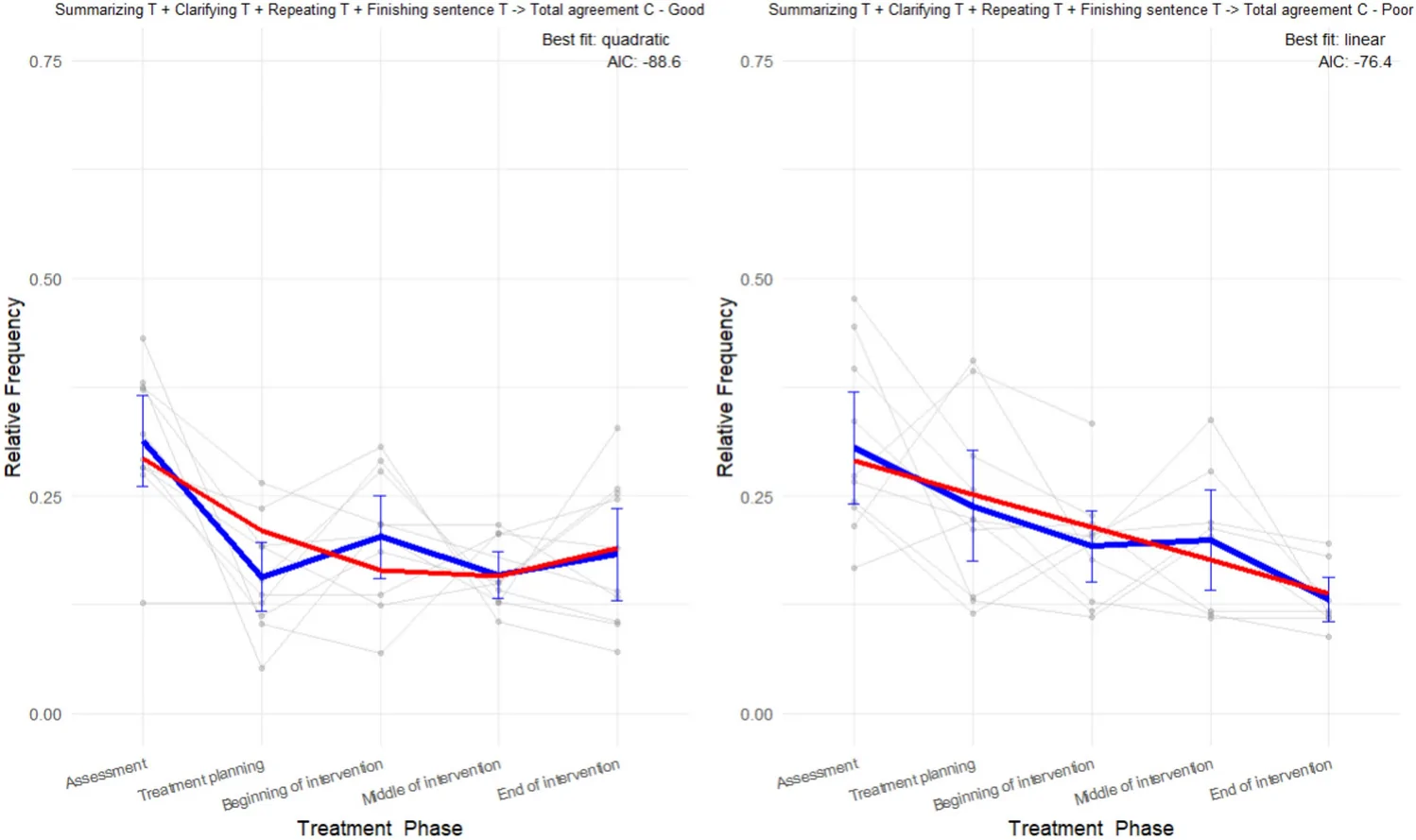
Plot generated with the function sequence_trajectory() comparing the empirical and predicted temporal means of the sequence “Active listening (therapist, various categories)—> Total agreement (client)” in the “Good” (*left panel*) and “Poor” (*right panel*) alliance groups. *Note: Note: T* = behavior emitted by the therapist; *C* = behavior emitted by the client. *Grey lines* = individual empirical frequencies; *blue line* = empirical mean; *red line* = model-implied mean. *Error bars* represent 95% confidence intervals, calculated as *M* ± *t*(.975, *n* – 1) × *SE*, where *n* is the number of dyads contributing data to that phase. The function sequence_trajectory() can alternatively compute bootstrap percentile intervals (ci_method = “bootstrap”)

The function sequence_trajectory() also allows for group comparisons through the group argument, making it possible to examine whether the shape of the change of *behaviors* differs between groups defined by the researcher. Figure 6 illustrates a comparison of one behavioral sequence across these “Good” and “Poor” therapeutic alliance groups. Section 3.3 of the supplementary website (https://dfernandezregueras.github.io/step-by-step-guide/) provides an example of how to use this functionality.

In Fig. 6, the sequence “*Active listening* (therapist) → *Total agreement* (client)” is plotted for the “Good” and “Poor” alliance groups (left and right panels, respectively). During the initial “Assessment” phase, both groups show their highest relative frequency of this sequence. This is expected, as a main objective of early sessions is for the therapist to understand the client’s problems and accurate summarization; reflected in the client’s total agreement, this is an effective strategy.

A notable divergence emerges as treatment progresses. In the Poor alliance group, the sequence declines linearly, reaching its lowest point at the “End of intervention” phase. In contrast, the “Good” alliance group shows an increase in this sequence during the final phase. This pattern suggests that therapists in the “Good” alliance group continue to engage in accurate active listening—particularly relevant as therapeutic goals are achieved and reflection becomes more central again—thereby facilitating client agreement. Conversely, therapists in the “Poor” alliance group may either reduce their use of active listening or provide less accurate summaries, resulting in lower client agreement. Therapists in the “Good” alliance group may concentrate active listening (and deliver it more accurately) at the beginning and end of therapy, when greater space for client input is available, while prioritizing other interventions in the middle phases. The estimated trajectory for the “Good” alliance group is quadratic, with an initial decline followed by and end-of-treatment increase. In contrast, the “Poor” alliance group follows a linear trajectory, marked by a continuous decline.

 As the most apparent differences between the groups in this behavior occur in the final parts of therapy, both researchers and therapists could make informed decisions about focusing on the final sessions in order to study them or modify their therapeutic practices (e.g., therapists could make an effort to be accurate in their summaries, focusing only on the information provided by the clients and avoiding uninformed or loose ones).

## Summary of contributions

 This work introduces a methodological framework for the quantitative analysis of interactive behaviors in dyads, organized around three key questions: *That behaviors are more frequent? How do behaviors evolve over time? Do changes in behaviors differ across groups?* We provide a suite of *R* functions for preprocessing, describing and visualizing observational data to address these questions.

These tools may enhance the understanding of psychological phenomena involving interdependencies between dyad members (Bateson & Martin, 2021; Bodner & Ceulemans, 2023; Hategeka et al., 2021), encouraging the broader use of systematic observation studies—an approach often underutilized due to time and resource demands (Atzil-Slonim et al., 2024; Robbins et al., 2024).

Acknowledging that some behaviors are inherently infrequent or context-dependent, despite their potential importance for therapeutic outcomes (Levy Chajmovic & Tishby, 2025), we offered: (1) a guide to preprocessing observational data from any coding software (e.g., The Observer XT); (2) procedures for computing and visualizing descriptive statistics; and visual tools for exploring (3) *behavior* frequency; (4) their evolution over time in a standardized metric; and (5) group differences in those trajectories across theoretically relevant groups.

## Theoretical and methodological implications

Our tools simplify the handling of systematic observational data, especially for analyzing behavior sequences. Traditionally, such analyses have focused on whether such sequences occur at rates greater than chance (Bakeman & Quera, 2011). The methods we propose offer novel ways to handle and represent sequential data, enabling new research questions and, consequently, new types of analyses. They are particularly useful for guiding exploratory analyses, allowing informed decisions about which treatment phases to focus on based on the prominence of specific *behaviors*.

Crucially, our contribution allows us to address a phenomenon highlighted in the literature and acknowledged by therapists: the importance of emitting certain behaviors at appropriate times (Levy Chajmovic & Tishby, 2025). Due to the relatively low frequency of these behaviors—compared to others that occur more regularly—they may have been overlooked under the existent research frameworks. For example, empathy in cognitive-behavioral therapy (CBT) is not as frequently emphasized, but it has been associated with positive outcomes (Hara et al., 2017). In Fig. 4, empathy is less frequent than disagreement-related behaviors; nevertheless, it remains an interesting focus to study given its importance in the literature. By standardizing phase-specific counts relative to each participant’s total occurrences of that behavior across all phases, we can access information that more frequent behaviors in specific parts of therapy might otherwise mask.

Importantly, although we focused on a clinical phenomenon—the therapeutic alliance—our approach and dedicated functions can be readily applied to other domains using systematic observation, such as educational and organizational psychology (Lehmann-Willenbrock, 2025; Zheng et al., 2024).

Furthermore, recent advances in large language models (LLMs) and artificial intelligence are poised to significantly lower the barriers to conducting observational research. These tools can facilitate resource-intensive steps such as transcription and coding of behaviors, substantially reducing the time required to work with observational data. As these capabilities become integrated into research workflows, systematic observation will become more feasible and scalable, enabling its use in larger and more diverse samples. Against this backdrop, the present contribution is particularly timely: by providing a framework for processing and exploring observational data, we offer methodological tools that can complement and extend AI-driven solutions, across a wide range of psychological research contexts.

## Applications beyond psychotherapy

Although our illustrative example comes from psychotherapy, the framework is designed for observational datasets in which dyadic or small group interactions are coded as event sequences with identifiers for session, agent, and behavior. This data structure is common, for example, in classroom research, where coding schemes capture student and teacher behaviors and their contingencies over time (e.g., the Revised Edition of the School Observation Coding System, REDSOCS; Jacobs et al., 2000). In such contexts, the proposed functions can be applied to questions such as how often a particular student behavior occurs (e.g., off-task interaction with peers), how such a behavior changes across instructional units, or whether the trajectories differ between teachers or interventions. Adapting the framework mainly requires mapping the existing codes to the generic *Behavior*, *Session*, *Agent*, and *Group* variables used by our functions.

Romantic relationship research provides another domain for dyadic observational coding. For example, the Brief Romantic Relationship Interaction Coding Scheme (BRRICS; Humbad et al., 2011) codes both individual partners’ affect and dyadic interaction patterns in videotaped couple discussions. In such datasets, our framework could be used to examine the frequency and trajectories of couple behavior sequences (e.g., criticism followed by withdrawal) across tasks or time points, or to compare these trajectories across clinically distressed versus non-distressed couples. As with educational applications, some adaptation of the coding scheme to the generic event-based format required by our functions would be necessary, but no changes to the underlying algorithms are required.

## Limitations and future directions

The tools presented in this work are intended as a necessary first step for analyzing observational data from interacting dyads. As such, we focused on descriptive tools to explore the frequency and distribution of the behaviors of interest. This information, although highly relevant for understanding interactive dynamics, can be complemented by subsequent analyses. Future work should, for example, offer additional procedures to enable longitudinal analyses that capture relations between observed behaviors and post-session self-reported questionnaire data, while accounting for their temporal interactions. Interested readers can already build on established sequential-analysis approaches—such as lag-sequential analysis—for conducting inferential tests on dyadic sequence data once it has been preprocessed and visualized using the workflow we propose (e.g., Bakeman & Quera, 2011). Designs with substantially more measurement occasions per dyad would additionally support hidden Markov, stochastic differential equation, or network-based models, which we see as a natural extension of the present framework.

A further consideration is that, in the illustrative example, the Good and Poor alliance groups were defined using the WAI, a self-report measure of alliance quality, whereas the behavioral records were derived from TRCS observational coding. Because both instruments tap related but not identical aspects of the therapeutic relationship (perceived alliance quality and observable alliance-facilitating behaviors), this example should be interpreted as evidence of convergence rather than as an independent test of construct separation. This convergence is nonetheless very informative, since agreement between perspectives on the alliance is typically moderate at best (Tryon et al., 2007), and correspondence between a self-reported classification and an observationally coded sequence was therefore not guaranteed by the grouping criterion. Beyond this convergence, the observational approach yields information that self-report instruments cannot provide: the WAI produces a global, retrospective judgment of alliance quality, whereas sequential analysis of TRCS codes identifies which specific therapist behaviors precede which client responses and how those contingencies change across phases of the intervention. Our framework enables future research to explore whether these behavioral contingencies add predictive or incremental value over WAI scores, for example by explaining additional variance in alliance ratings or in treatment outcome.

One additional limitation when working with small numbers of dyads is the potential for observing longitudinal patterns that reflect chance variations due to the unique characteristics of those dyads. To address this, we implemented regularized Bayesian linear mixed effects models (BLMMs) in the function sequence_trajectory() for more stable estimates. For the same reason, the confidence intervals displayed around phase means are computed from the *t* distribution rather than the normal approximation, and a bootstrap alternative is available for phases in which the assumption of normality is untenable. Still, researchers should interpret results in light of their theoretical and domain-specific knowledge and avoid overgeneralizing from descriptive outputs alone. 

Recent advances in large language models (LLMs) and related AI technologies are likely to make observational research far more practical in the coming years. These tools can automate tasks that have traditionally limited sample sizes, such as transcription, behavior coding, and preliminary identification of interaction patterns. However, their use remains preliminary, with limitations including hallucinations, context-window constraints, and limited validation against human coders for nuanced affective or interactional judgments (Deng et al., 2023). By reducing the time and resources required observational studies, LLMs will enable researchers to scale them up and apply them in diverse contexts. This anticipated shift underscores the importance of developing transparent and reproducible frameworks, such as the one we present here.

## Conclusion

In this work, we presented a framework for describing interactive behavioral patterns obtained through systematic observation. The proposed procedures underscore the importance of timing and context—especially for infrequent yet meaningful behaviors. By enabling preprocessing, visualization, and normalized longitudinal analysis, our tools support richer, theory-driven research across domains centered on interpersonal dynamics.

## Data Availability

A dedicated website was created to organize a step-by-step application of the procedures described in this article. Materials, data, and the *R* code used in this manuscript are publicly available, in compliance with a type 4 research data policy, at https://dfernandezregueras.github.io/step-by-step-guide/

## Declarations

### Open practices statement

The reported study was not preregistered. Materials, data, and the *R* code used in this manuscript are publicly available at https://dfernandezregueras.github.io/step-by-step-guide/
